# *Metarhizium brunneum* infection dynamics differ at the cuticle interface of susceptible and tolerant morphs of *Galleria mellonella*

**DOI:** 10.1080/21505594.2019.1693230

**Published:** 2019-11-25

**Authors:** Ekaterina V. Grizanova, Christopher J. Coates, Ivan M. Dubovskiy, Tariq M. Butt

**Affiliations:** a Laboratory of Biological Plant Protection and Biotechnology, Novosibirsk State Agrarian University, Novosibirsk, Russia; b Department of Biosciences, College of Science, Swansea University, Swansea, UK; c Siberian Federal Scientific Centre of Agro-BioTechnologies, Russian Academy of Sciences, Krasnoobsk, Russia

**Keywords:** Innate immunity, host-pathogen interactions, cuticle, entomopathogenic fungi, melanization, *Galleria mellonella*

## Abstract

In order for entomopathogenic fungi to colonize an insect host, they must first attach to, and penetrate, the cuticle layers of the integument. Herein, we explored the interactions between the fungal pathogen *Metarhizium brunneum* ARSEF 4556 and two immunologically distinct morphs, melanic (M) and non-melanic (NM), of the greater wax moth *Galleria mellonella*. We first interrogated the cuticular compositions of both insect morphs to reveal substantial differences in their physiochemical properties. Enhanced melanin accumulation, fewer hydrocarbons, and higher _L_-dihydroxyphenylalanine (DOPA) decarboxylase activity were evident in the cuticle of the M larvae. This “hostile” terrain proved challenging for *M. brunneum –* reflected in poor conidial attachment and germination, and elevated expression of stress-associated genes (*e.g., Hsp30, Hsp70*). Lack of adherence to the cuticle impacted negatively on the speed of kill and overall host mortality; a dose of 10^7^ conidia killed ~30% of M larvae over a 12-day period, whereas a 100-fold lower dose (10^5^ conidia) achieved a similar result for NM larvae. Candidate gene expression patterns between the insect morphs indicated that M larvae are primed to “switch-on” immunity-associated genes (e.g., phenoloxidase) within 6–12 h of conidia exposure and can sustain a “defense” response. Critically, *M. brunneum* responds to the distinct physiochemical cues of both hosts and adjusts the expression of pathogenicity-related genes accordingly (e.g., *Pr2, Mad1, Mad2*). We reveal previously uncharacterized mechanisms of attack and defence in fungal-insect antibiosis.

## Background

The sclerotized integument, or exoskeleton, of an insect provides the first line of defence against opportunistic and obligate pathogens. This robust, multi-layered, physiochemical barrier is composed of biocidal epicuticular fatty acids and a protein-chitin procuticle reinforced with melanic polymers [1,2]. Hypocrealean entomopathogenic fungi (EPF) such as *Metarhizium brunneum* and *Beauveria bassiana* have evolved adhesion factors, hydrolytic enzymes, and specialized infection structures to invade the cuticle directly [3,4]. These unique features contribute invariably to their success as biocontrol agents – providing an environmentally friendly alternative to chemical pesticides that have been withdrawn from the market or to which pests have developed resistance [5].

The physiochemical properties of the cuticle can interfere with different facets of the fungal infection process which, in turn, impacts specificity and virulence [3]. For example, the surface chemistry can influence the adhesion of inocula due to weak adhesion forces [6,7] or fungistasis [3,8]. Sequestration of fungistatic plant allelochemicals by the epicuticular waxes can also impede infection [3]. Delaying penetration of the cuticle predisposes the fungal inoculum to other biotic and abiotic factors that are deleterious to the pathogen. Such factors include low or fluctuating humidity, rainfall (which washes off spores), and UV radiation [9–11]. Thus, successful EPF strains are not only able to cope with the physical environment of the host surface but also the preformed innate immune defences of the insect. Beneath the cuticle, the epidermis synthesizes antimicrobial peptides and activates the stress management apparatus [12,13]. These front line defenses are augmented with the humoral and cellular responses within the hemolymph, some of which follow the gradient of cues emanating from the fungus or infection site [14,15]. The innate immune responses within the hemocoel (body cavity) of the host have received comparatively more attention than the cuticle, in spite of the latter being the primary and most important barrier to disease-causing agents.

Larvae of the greater wax moth, *Galleria mellonella*, are used frequently as *in vivo* models for assessing the virulence of disease-causing agents, e.g., entomopathogens [16,17], and identifying the biological targets of toxins [18]. In 2013, a distinct melanic (darker) morph of *G. mellonella* was discovered, which demonstrated enhanced resistance to the EPF *B. bassiana* relative to the “normal” non-melanic morph [12]. Having access to both tolerant (melanic; M) and susceptible (non-melanic; NM) morphs of *G. mellonella* further enhances its usefulness as an experimental tool for deciphering the molecular mechanisms that underpin the attack and counterattack strategies of entomopathogens and insects, respectively. The overall aim of this study was to characterize the interactions between a virulent strain of *M. brunneum* and the distinct morphs of *G. mellonella* during the initial, critical stages of infection. To achieve this, we first assessed the preformed defenses, i.e., immune gene expression and physiochemical (melanization, hydrocarbon, fatty acid) contents, of naive M and NM morphs. Second, we exposed larvae to *M. brunneum* and recorded conidial attachment to, and germination on, the cuticle of each morph as well as differential expression of insect genes encoding immune factors (e.g., apolipophorin III), stress management (e.g., HSP90) and detoxification (e.g., glutathione-S-transferase). Third, we surveyed fungal gene expression across key stages of insect colonization: adhesion to the cuticle (*Mad1, Mad2*), cuticle degradation (*Pr1, Pr2*), stress management (*Hsp30, Hsp70*), differentiation of infection structures (*cag8*), and nutrient assimilation (*nrr1*).

## Results

### Biochemical and biophysical properties of melanic versus non-melanic integuments

**Figure 1.**
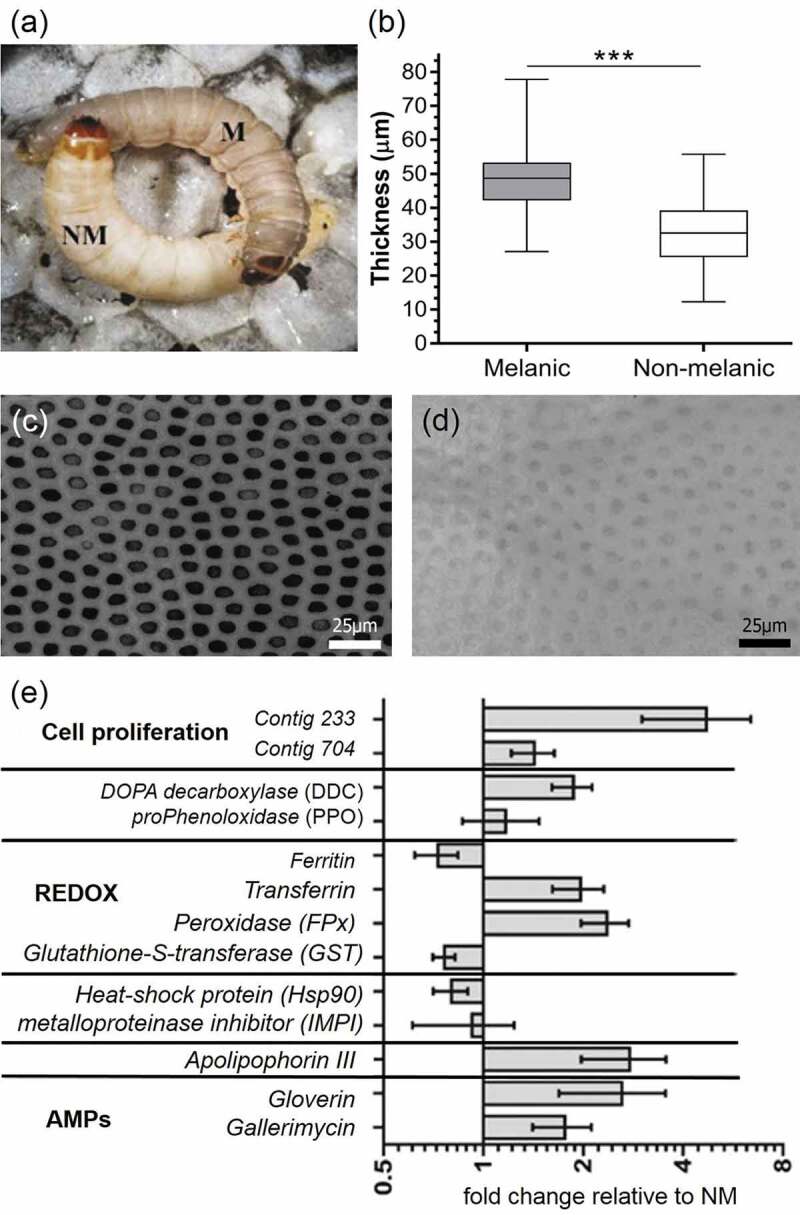
**Integument properties and candidate gene expression of *Galleria mellonella* morphs**. Appearance (a), cuticle thickness (n = 170, *** = p < 0.001), and patterns of melanin deposition (c, d) of melanic and non-melanic morphs. Enhanced expression (mRNA levels) of genes encoding immune factors (gloverin, gallerimycin, apolipophorin III, IMPI, DDC, PPO), stress management (HSP90), detoxification, and cell proliferation of naive melanic (M) larvae compared to non-melanic (NM) larvae (e).

The integument of melanic (M) *G. mellonella* are visibly darker in colour compared to the non-melanic (NM) morph (Figure 1(a)), and ~1.5-fold thicker (Figure 1(b); p < 0.001, t = 15.18 df = 348). This distinct colouration is due to the M insects having ~1.8-fold more surface covered in melanic spots (p < 0.001, t = 6.058, df = 18), with each spot ~14.7 times darker than their NM counterparts (p < 0.001, t = 79.72, df = 18). Additionally, the non-melanized distance between each spot measures 4.66 ± 0.15 µm and 7.25 ± 0.31 µm on M and NM larvae, respectively (p < 0.001, t = 7.485, d f = 18; Figure 1(c,d)).

**Figure 2.**
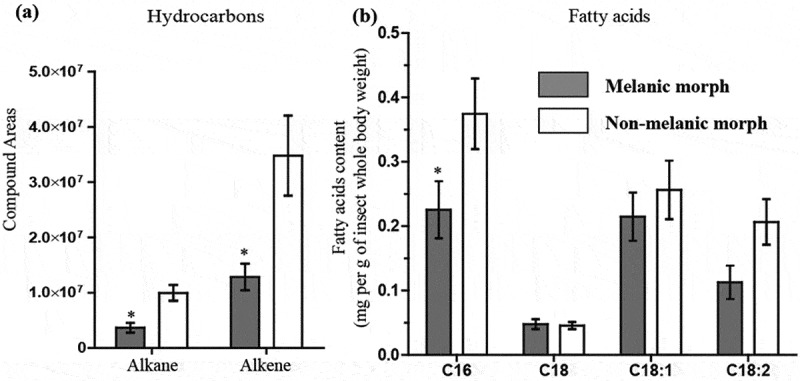
**Biochemical profiles of *Galleria mellonella* morphs.** Quantities and compositions of epicuticular hydrocarbons (a) and fatty acids (b) from un-stimulated (i.e., naive) melanic and non-melanic wax moth larvae. Unpaired t-tests were used to assess differences between each insect morph (* = p < 0.05).

An initial survey of candidate gene expression between un-stimulated (non-infected) M and NM insects revealed key differences in “immune-vigor”. Levels of mRNA for antimicrobial peptides (gallerimycin, gloverin) and the multi-functional immune-factor apolipophorin III were two- to threefold higher in M larvae (Figure 1(e)). Several genes linked to reduction/oxidation (REDOX) management (e.g., peroxidase), eumelanin biogenesis (e.g., DOPA-decarboxylase), and growth factors (1.5-fold for *contig704* and fivefold for *contig233*) in the integument were elevated in M larvae (Figure 1(e)). Using a preliminary annotation of the recently published *G. mellonella* genome [19], we identified *contig704* to be a pleiotrophin-like protein precursor. Phenotypic differences between insect morphs were reflected in the biochemical compositions of the integuments. Epicuticular extracts of M larvae contained 2.7–2.9 fold fewer alkanes/alkenes (p < 0.05, t = 3.745, df = 4) and hydrocarbons (p < 0.05, t = 2.877, df = 4) compared to their NM counterparts (Figure 2(a)). Melanic larvae also had significantly reduced numbers of C16 fatty acids (p < 0.05, t = 2.74) (Figure 2(b)). The C18, C18:1 and C18:2 contents appeared similar between the two insect morphs.

### *Differential susceptibility of* Galleria Mellonella *morphs to* Metarhizium brunneum

**Figure 3.**
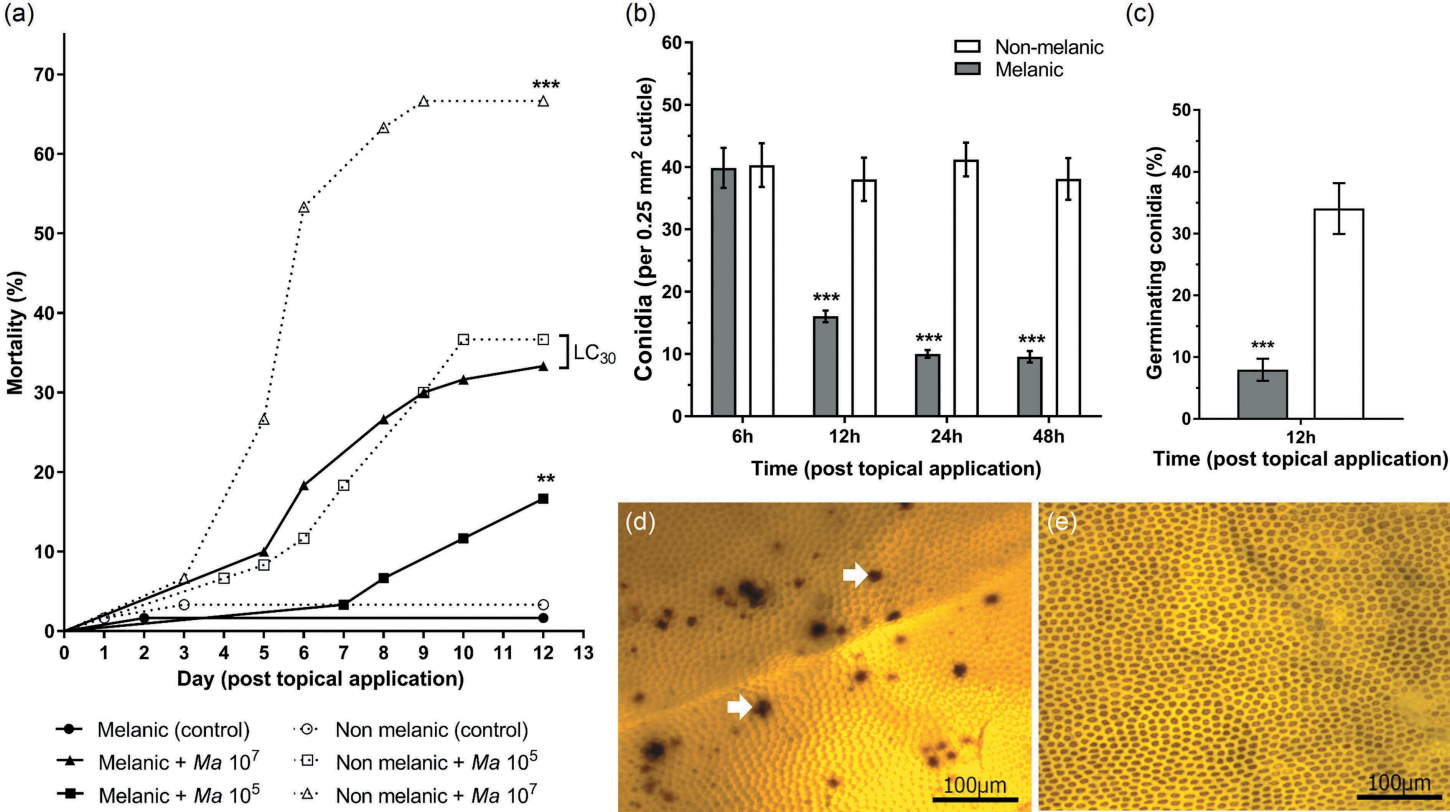
**Development of mycosis (*Metarhizium brunneum)* in melanic (M) and non-melanic (NM) morphs of *Galleria mellonella* larvae after topical inoculation**. The survival of each insect morph was recorded over 12 days following exposure to two doses of *M. brunneum* conidia. LС_30_ values demonstrate similar mortality levels of M and NM larvae in the presence of 10^7^ conidia (p < 0.01, **) and 10^5^ conidia (p < 0.001, ***), respectively (*n* = 60). The numbers of conidia per 0.25 mm^2^ cuticle were counted on M and NM morphs during infection with *M. brunneum* (10^7^) (b), and the percentage of germinating conidia (c) that successfully adhered (*** = p < 0.001; n = 22). Penetration of the integument by germinating conidia led to the formation of melanotic lesions (white arrows) on NM larvae – representing *de novo* synthesis of melanin (d). Conversely, the pattern of melanin deposition on M larvae remained unchanged (e), indicating the melanic-defenses are preformed (images were taken 24 h.p.i. with 10^7^ conidia).

Topical exposure of *G. mellonella* morphs to two doses of *M. brunneum* conidia (1 × 10^5^ and 1 × 10^7^) led to significant decreases in survival over a 12-day period (Figure 3(a); *X*^2^(5) = 108.5, p < 0.0001). The highest mortality of 67% was observed for NM larvae exposed to 1 × 10^7^ conidia of *M. brunneum*. Moreover, NM larvae were significantly more susceptible to either fungal dose (*p* = 0.0083 (1 × 10^5^), *p* < 0.0001 (1 × 10^7^)). The lower dose (1 × 10^5^) of conidia was sufficient to kill ~36% of NM larvae, whereas the higher dose (1 × 10^7^) was required to kill ~33% of the M larvae (Figure 3(a)). A resistance ratio (RR) calculation indicated M larvae are 31-fold less sensitive to *M. brunneum*. Mortalities for uninfected (control) insects were ≤3.3% for either insect morph.

Adhesion of *M. brunneum* conidia to the integuments of M and NM larvae were almost identical (~40%) within 6-h post-topical application (Figure 3b). Over the subsequent 12–48 h, ~75% of the conidia dropped-off the M larvae, and <8% of those remaining showed signs of germination (Figure 3b, p < 0.001). Germination of conidia on the living insects following fungal application were fourfold higher at 12-h post-infection on the NM larvae (Figure 3(c); t = 14.71, p < 0.001). At 24 and 48 h, conidial germination on M larvae were 37% and 71%, respectively, compared to 100% on NM larvae (t = 14.03 and t = 6.53, p < 0.001; Supplementary Figure 1). The formation of melanic lesions caused by multiple penetrating fungi were clearly visible on the integument of NM larvae (Figure 3(d)). Conversely, no clear signs of additional melanin deposition were seen on M larvae in response to the pathogen over the same experimental period (Figure 3(e)).

### Expression of insect immunity/stress-related genes in the integument

**Figure 4.**
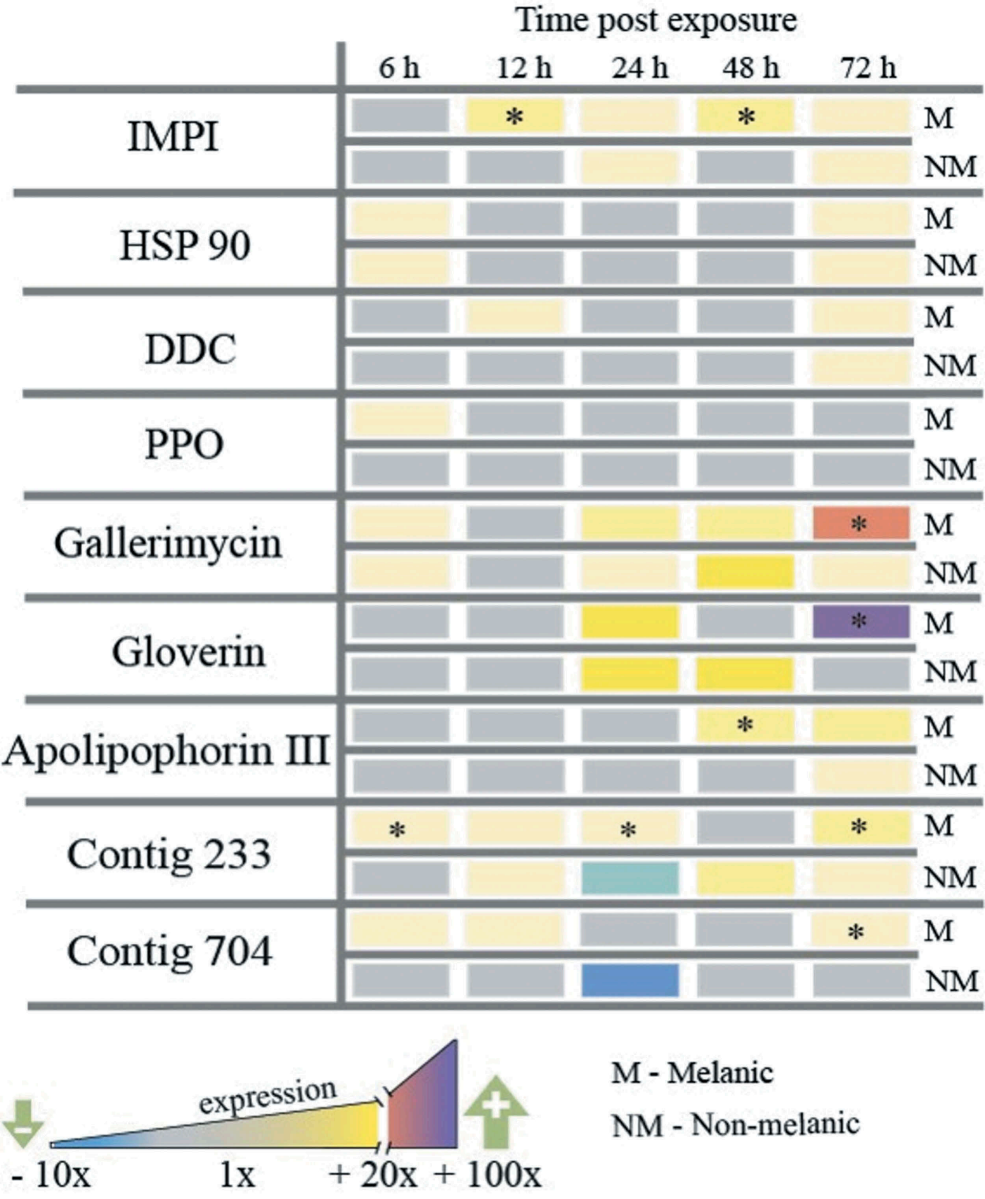
**Gene expression (mRNA levels) of *Galleria mellonella* integumental tissues exposed to *Metarhizium brunneum* conidia (LС_30_) relative to uninfected (control) insects**. Insect metalloproteinase inhibitor (IMPI), heat-shock protein (HSP 90), DOPA decarboxylase (DDC), prophenoloxidase (PPO), antifungal peptide gallerimycin, antibacterial peptide gloverin, β-glucan binding protein apolipophorin III, growth factors contig 233 (growth-blocking peptide) and contig 704 (pleiotrophin-like protein). (* = p < 0.05; melanic versus non-melanic at the respective time point).

Exposure of M larvae to *M. brunneum* via topical application stimulated the rapid (6 – 12 h) up-regulation of several key genes encoding anti-infective factors in the integument: fivefold increases in the insect metalloproteinase inhibitor protein (*IMPI* [20]), the antifungal peptide gallerimycin, and the melanin-generating enzymes DOPA-decarboxylase and proPhenoloxidase (Figure 4). Elevated expression of genes encoding two putative growth factors, *contig233* and *contig704*, and the stress-associated protein chaperone, *heat shock protein 90* (*Hsp90*), were observed also in M larvae within 12 h. Conversely, NM larvae that were exposed to the same dose of *M. brunneum* contained increased mRNA levels for *Hsp90* and *gallerimycin* only (at 12 h). By 72 h post-infection, M larvae increased the expression of *gallerimycin* and the gene encoding the antibacterial peptide *gloverin* by 38-fold and 24-fold, respectively (Figure 4). Regarding the multifunctional β-glucan-binding protein apolipophorin III, M larvae switched this gene on earlier at 48 h compared to 72 h for NM larvae, and overall, produced sixfold more mRNA.

**Figure 5.**
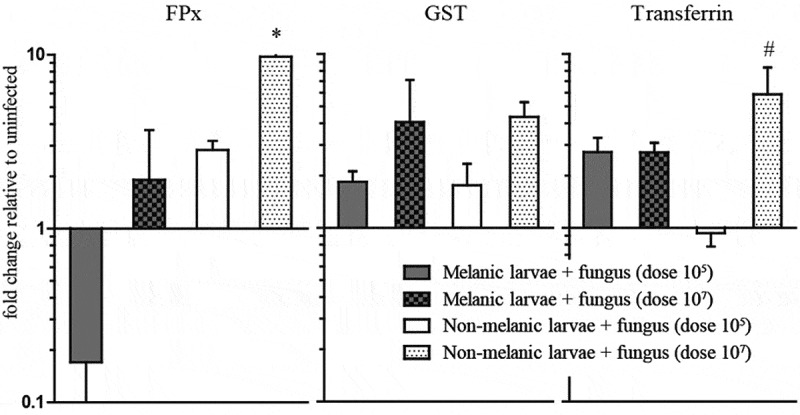
**Detoxification-associated gene expression in *Galleria mellonella* morphs exposed to *Metarhizium brunneum***. Antioxidant enzymes, peroxidase (FPx) (a), glutathione-S-transferase (GST) (b) and transferrin, (c) in the integuments of M and NM larvae at 72 h post fungal infection. The asterisk [*] indicates a significant difference (p < 0.05) between NM (10^7^) and M (10^5^). The hashtag [#] indicates a significant difference (p < 0.01) between NM (10^7^) and NM (10^5^).

Messenger RNA levels of the antioxidant enzymes, peroxidase (FPx) and glutathione-S-transferase (GST), increased in a dose-dependent manner across 72 h in the integuments of both M and NM larvae (Figure 5). Differential expression patterns were higher in the NM, and were perhaps linked to the *de novo* synthesis of melanin at the sites of fungal penetration (Figure 3e). Expression of the siderophore transferrin also increased with inoculation dose in the NM, but this was not the case for the M larvae (Figure 5).

### *Expression of* Metarhizium brunneum *virulence/stress-related genes on the insect integument*

**Figure 6.**
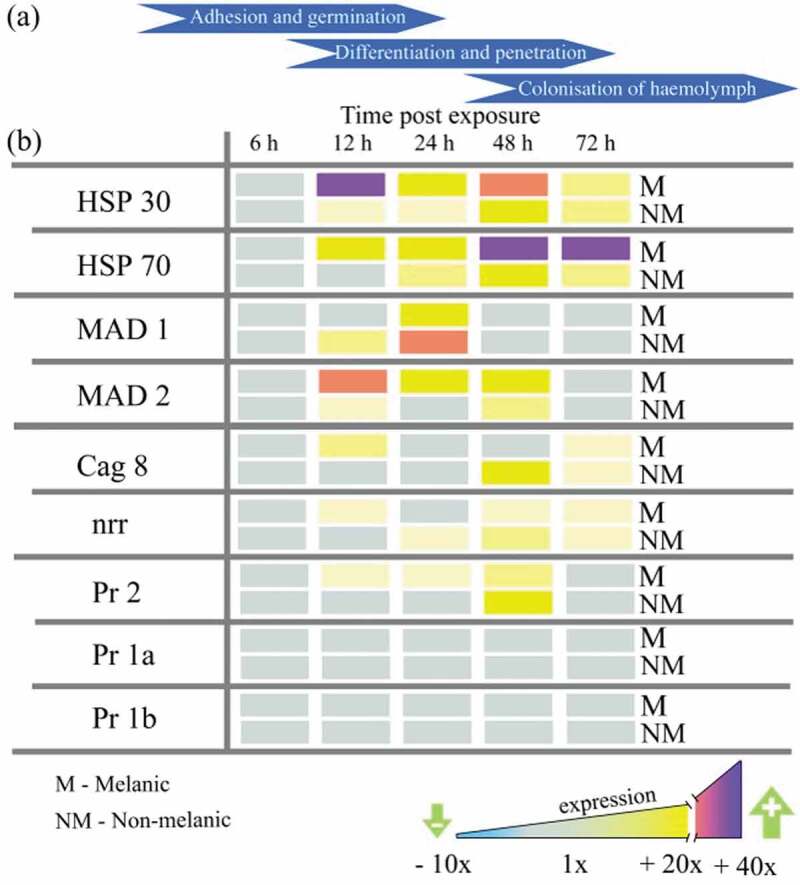
**Gene expression (mRNA levels) of *Metarhizium brunneum* during the infection cycle on melanic and non-melanic morphs of *Galleria mellonella***. Scheme representing the broad stages of host colonization (a). Subtilisin-like proteases (*Pr1a, Pr1b, Pr2*), heat-shock proteins (*HSP30, HSP70*), adhesin-like proteins (*MAD1* and *MAD2*), conidiation – associated gene (*cag 8*) and nitrogen regulator response (*nrr*) expression on the insect integuments from 6 to 72-h post-exposure.

**Figure 7.**
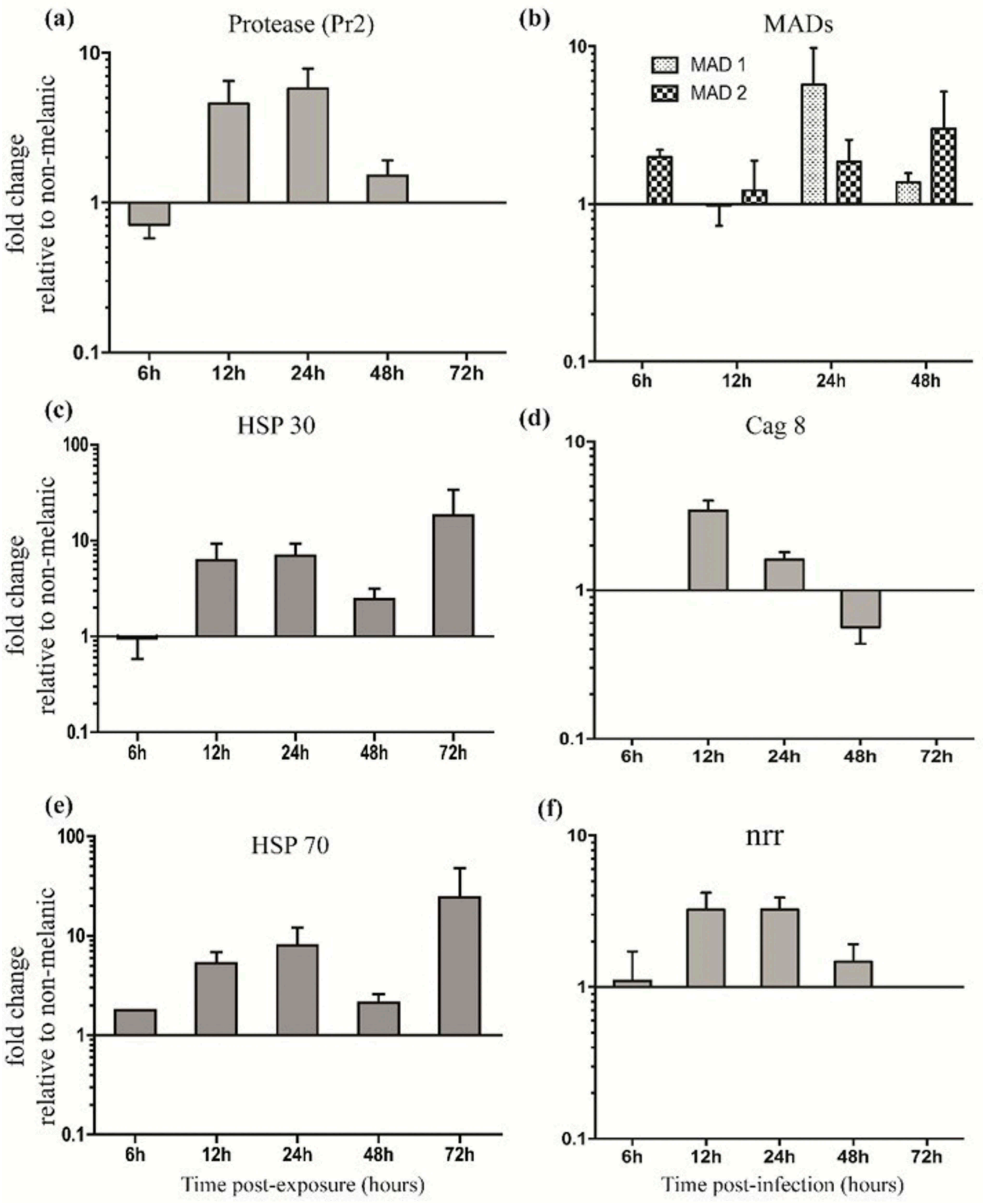
**Relative expression patterns of *Metarhizium brunneum* genes on the integuments of melanic larvae compared directly to non-melanic larvae**. Fungal protease *Pr2* (a), adhesin-like proteins (*MAD1* and *MAD2*) (b), heat-shock proteins 30/70 (*HSP30/70*, c/e), conidiation-associated gene (*cag 8*, d) and nitrogen regulator response (*nrr*, f).

*Metarhizium brunneum* gene expression on the integument indicated a host-dependent response, with adhesion (*Mad2*), stress management (*Hsp30, Hsp70*), differentiation of infection structures (*cag8*), nutrient assimilation (*nrr1*), and cuticle degradation (*Pr2*) genes all up-regulated significantly (5x to 25x) after 12 h on M larvae (Figure 6(a,b)). Fungi infecting the NM larvae switched-on the insect-specific adhesion factor *Mad*1 (sixfold), whereas, those infecting the M larvae expressed the generalist (plant-specific) *Mad2* (20-fold; Figure 6(b), Figure 7). Moreover, increased expression of protease *Pr2* ranged from 2- to 10-fold between 12 and 48 h, with *cag 8* and *nitrogen regulator (nrr1)* mRNA increasing from 2- to 6-fold between 12 and 72 h (Figure 6(b)).

The expression of fungal virulence/stress management genes on M and NM integuments using the same lethal concentration (LC_30_; Figure 3(a)) showed that the protease gene *Pr2* was upregulated 4.5 and 5.7-fold at 12 h and 24 h (Figure 7(a)). Surprisingly, differential expression of the other subtilisin-like (cuticle-degrading) protease genes *Pr1a* and *Pr1b* was not detected on either M or NM larvae across the 72 h period (Figure 6(b)). Levels of *M. brunneum* heat-shock proteins (*Hsp 30* and *Hsp 70)* were 5 to 25-fold higher on M integuments compared to NM at each time point from 12 h onwards (Figure 7). Expression of the fungal adhesin *Mad1* was sixfold higher in NM larvae at 24 h. *Mad2* expression in M larvae was consistently higher. The Cag8 and nitrogen regulator (*nrr*) mRNA levels were threefold higher in integuments of M insects at 12 h and 24 h pi compared to NM insects (Figure 7(d,f)).

## Discussion

In this study, two morphs (melanic (M) and non-melanic (NM)) of *G. mellonella* were used successfully to interrogate *M. brunneum*-insect antibiosis at the cuticle interface. Firstly, our data strengthen earlier findings that the two insect variants are indeed immunologically discrete [12]. The cuticle of the M morph is thicker, replete with melanic deposits that limit colonizable area, and contains significantly fewer hydrocarbons and fatty acids. These features describe a rather inhospitable surface to which *M. brunneum* could adhere to, thereby resulting in prolonged exposure of the fungus to environmental stress (e.g., UV) and nutrient limitations. In contrast to NM larvae, and in the absence of infection, the M cuticle has higher basal levels of specific antifungal factors (apolipophorin III, gallerimycin, IMPI), antibacterial factors (gloverin) and general defenses (DOPA-decarboxylase, transferrin). Therefore, the M larvae are primed/front-loaded to tackle diverse disease-causing agents in contrast to their NM counterparts. This observation is supported by the retarded infection process displayed by *M. brunneum* on the M larvae when compared to the NM; fewer conidia adhere to and germinate on the cuticle, distinct up-regulation of stress genes (*Hsp30, Hsp70*), and it takes 100-times more fungi to reduce larval survival by 30%.

Delaying the fungus at the surface of the cuticle provides the M larvae with more time to mobilize hemolymph defenses should the fungus eventually gain entry into the hemocoel (body cavity). Cuticular penetration of M larvae by the entomopathogen, *B. bassiana*, was subdued by rapid cellular (hemocyte) encapsulation [12]. This was not the case when *B. bassiana* was applied to M larvae using intrahemocoelic injection – indicating that signals from the cuticle are relayed to the hemolymph. Mukherjee and Vilcinskas [14] recently discovered that *Metarhizium robertsii* could sense distinct antimicrobial peptides and protease inhibitors derived from insects (e.g., *G. mellonella*), and respond by producing specific chymotrypsin-like and metalloproteinases to neutralize the host molecules. The intensity of this response can be attributed to the virulence of each fungal strain, and is regulated epigenetically during infection. A “good” entomopathogen colonizes and then compromises the host without killing (i.e., immune-suppression and/or immune-evasion) – thereby monopolizing the insect resources. In turn, the insect unleashes a battery of immune effectors to recognize and immobilize the fungal threat (e.g., apolipophorin III [21];). Fungi-stasis can be achieved by weaponizing melanin through phenoloxidase activity [22] and is accompanied by fungicidal compounds (e.g., gallerimycin). Insects also release proteinase inhibitors, e.g., IMPI [20], siderophores and detoxifying agents (e.g., transferrin, glutathione-s-transferase [23]) in order to thwart the fungus. The co-evolution of these attack and defense strategies has led to an intimate gene-for-gene relationship between certain EPF and insects, in addition to episodes of molecular diversification to broaden host ranges [24]. Herein, our data support both observations that insects and their would-be colonizers communicate at the cuticle interface, and activate antagonistic (targeted) gene products throughout the various stages of infection.

The quantity and distribution pattern of melanic deposits in M larvae appeared to interfere with fungal adhesion and germination. As insect mortality is dependent on the conidial dose [9], this could account for the differences in susceptibility for M and NM larvae. Initially, high numbers of conidia attached to both M and NM insects. This suggests that the first, passive adhesion step entailing hydrophobic interactions was unaffected. However, the second step, which involves the production of enzymes and mucilage to consolidate adhesion, was disrupted since the number of conidia adhering 12 h post-inoculation declined dramatically on M insects. Most often, a decline is attributed to fungistatic compounds in/on the cuticle, as reported for the flea beetle *Psylliodes chrysocephala* [3,8]. However, other aspects of the pathogen-cuticle interaction cannot be ignored, especially those influencing the secretion of cuticle-degrading enzymes such as ambient pH and cuticle peptide digest products [3, 25–27]. The numbers of conidia on M cuticle at 24 h and 48 h remained intact, indicating they had consolidated attachment and represent the population of conidia that landed on the more conducive, non-melanized, regions. We posit that melanization interfered with *M. brunneum* conidia attachment but there are many examples of EPF adhering to and infecting insects with highly melanized cuticles [3]. However, differences in conidia adhesion could also be linked to the alternate expression of adhesins, *Mad1* and *Mad2*. The insect cuticle-specific *Mad1* adhesin was expressed sooner on NM than M, peaking at 24 h with significantly higher levels of transcripts being observed on NM. In contrast, *Mad2*, which is linked with conidial attachment to plants [26], was highly expressed at 12 h on M insects and produced for longer when compared to the NM hosts. Barelli *et al*. (2011) [27] found that *Mad2* was up-regulated in *M. robertsii* by nutrient starvation but not oxidative or osmotic stress. In contrast, *Hsp30* and *Hsp70* were up-regulated by oxidative stress even under nutrient-rich conditions [28]. The genes associated with the development of infection structures (*cag8*) and nutrient assimilation (*nrr*) were highly expressed on M larvae at 12 h pi, demonstrating that *M. brunneum* ARSEF 4556 was responding to different cues and presumably implementing different infection strategies [28,29].

Besides poor adhesion, infection of M larvae would be slower because melanized cuticles are harder to degrade [30]. Melanic polymers protect against enzyme degradation and liberation of nutrients (i.e., steric hindrance). The cuticle-degrading proteases *Pr1* and *Pr2* release melanin from cuticles by hydrolysis of the associated protein [31]. Pr1 is a major cuticle degrading enzyme expressed by EPF in response to cuticle and nutritional cues with the Pr1a being the dominant of eleven (a-k) isoforms [32,33]. Surprisingly, *Pr1a* and *Pr1b* expression were not detected here but *Pr2* levels were elevated on both M and NM cuticles. It is possible that *M. brunneum* ARSEF 4556 used here lacks the genes encoding *Pr1*, as previous work has characterized the presence of spontaneous *Pr1a/b* deficient mutants within wild-type *Metarhizum* spore populations [34]. We presume that the expression of *Pr2* is intended to compensate for Pr1 deficiencies during the first critical 48 h. However, we cannot rule out the likely possibility that other virulence factors, such as chymotrypsin-like proteinases and metalloproteinases, compensate for *Pr1* absence (especially as we detected the elevated expression of IMPI on infected *G. mellonella*). The M cuticle did induce elevated Pr2 levels earlier than the NM cuticle demonstrating the ability of the fungus to implement different attack strategies in response to surface chemistry cues. The capacity for *M. brunneum* to compensate for a decline in Pr1 was reported for a Pr1-deficient mutant [34]. This mutant, although less virulent overall than the wild type, was more virulent for wax moth larvae than the more melanized mealworm larvae [34]. Rosas-Garcia *et al*. (2014) [35] reported variations in the expression of Pr1 and Pr2 and suggested that these enzymes are pathogenicity (not virulence) determinants. They found the most virulent strain of *M. anisopliae* for *Spodoptera exigua* was one that produced the most Pr2. Similarly, Golo *et al.* (2015) [36] could not establish a relationship between Pr1 and virulence of *M. anisopliae* against ticks. These findings are contrary to those of other workers who found Pr1 being an important virulence determinant [37]. Altogether, these observations suggest that strains of EPF have diverged as to which enzymes to deploy when infecting different hosts.

Alkane, alkene and fatty acid hydrocarbons at the surface of the insect cuticle can influence spore attachment, germination, and viability [3,38]. Crespo *et al*. (2002) [39] found that some alkanes enhanced fungal virulence, while Jarrold et al. (2007) [40] suggest that simple polar compounds at the cuticle surface may be required to stimulate germination before the entomopathogenic fungus can utilize more complex blends of nonpolar lipids. Herein, the relatively low quantities of hydrocarbons at the surface of the M cuticle may represent a nutrient-restricted environment. This would require less investment of energy than insects dependent on the secretion of copious amounts of antifungal compounds. Interestingly, an earlier study showed that the *G. mellonella* cuticle lacked fungistatic fatty acids [41].

Heat shock proteins 30 and 70 play pivotal roles in stress management during insect, plant and vertebrate mycosis [6,42,43]. Both heat shock proteins were expressed in *M. brunneum* ARSEF 4556 from 12 h onwards with levels being significantly higher on M larvae. The expression coincides with the second, active phase of conidia adhesion (12 h pi) with another burst of activity during penetration and colonization (72 h pi). It is likely that the fungus was responding to different stresses. The initial peak, especially *Hsp30*, is likely due to nutritional and oxidative stress [27]. High levels of reactive oxygen species are produced by the host, which triggers a concomitant antioxidant response by the fungal pathogen [44,45]. Heat shock proteins can limit damage and may explain why they were elevated in both M and NM larvae. The enhanced expression of *Hsp70* by *M. brunneum* at 12 h on M larvae during the critical penetration and colonization phase likely reflects the prolonged attempt to overcome the reinforced cuticle of the M morph as outlined above.

Generally, the up-regulation of immune genes was faster in M than NM larvae demonstrating that signalling is not impaired by either cuticle thickness or degree of melanization. However, differences in the temporal expression of immune and stress management genes likely reflect differences in the “translation” of these signals. For example, DOPA-decarboxylase activity was twofold higher in M larvae and partially accounts for the eumelanin load observed. In insects, _L_-dihydroxyphenylalanine (_L_-DOPA) is an endogenous substrate of phenoloxidase enzymes in the hemolymph, however, DOPA-decarboxylase converts _L_-DOPA into dopamine, which tends to be re-directed to the insect cuticle and oxidized by laccases (para-diphenoloxidase) into melanic polymers [22]. Dopamine itself can be a marker of stress during fungal infections with “bursts” of dopamine fuelling the development of local defense reactions of NM larvae [46]. When *M. brunneum* started to penetrate the cuticle of our M larvae there was no evidence of further release or *de novo* synthesis of the pigment. It is possible that M larvae either pre-allocate the maximum melanogenesis-associated resources into the cuticle, or, the toxic by-products of melanin precursors would exceed the tolerance levels of the host. Excessive levels of melanin and its precursor quinones are not only harmful to the invading pathogen but can prove lethal for the insect if they are not modulated [47–49].

If conidia manages to overcome the front-loaded melanin-associated defenses, they face higher levels of the anti-fungal peptide gallerimycin, and the multi-functional β-glucan binding protein apolipophorin III [21]. Antibacterial peptides such as gloverin would have a limited impact on EPF but deter the establishment of opportunistic saprophytic microbes [3,14]. Apolipophorin III enables *G. mellonella* larvae to discriminate between pathogens and mount an adequate cellular immune response [50].

During the hemocoel colonization phase (72 hpi), *M. brunneum* elicited a strong antioxidant (FPx, GST) response in wax moth larvae with activity being dose-dependent. The response was greater in NM insects than M larvae due to more rapid and higher infection – resulting in greater numbers of colonizing hyphal bodies. In contrast, transferrin levels increased with dose in the NM but not M larvae, reflecting a divergence in the regulation of this siderophore. As new regions of the NM cuticle became melanized during fungal invasion, it would be sensible to employ detoxification machinery to avoid collateral damage – an approach that has been recently reported in *G. mellonella* during hemocyte encapsulation [23].

The M morph of *G. mellonella* is evolutionarily primed to withstand EPF and can survive doses of *M. brunneum* and *B. bassiana* that are lethal to NM larvae. Such immune-vigor does have trade-offs. In our previous study, we characterized reduced fecundity in M larvae compared to NM, but markedly higher resistance to *B. bassiana* [12]. Selective breeding of the M morph of *G. mellonella* over 25 generations led to enhanced resistance against *B. bassiana –* characterized by higher PO activities and expression of defense genes in the integument [13]. High levels of contigs 233 and 704 suggest that cell proliferation and regeneration in M insects could help repair the sites of fungal penetration faster than the average NM larva. Therefore, the M larvae are quick to respond to microbial attack and quick to heal/repair.

### Concluding remarks

Our work has taken advantage of a previously reported distinct (melanic) morph of *G. mellonella*, and examined whether melanization plays a vital role in anti-infective defense of insects and how parasitic fungi cope with hosts differing in susceptibility to microbial attack. We provide strong evidence that the entomopathogenic fungus, *M. brunneum* ARSEF 4556, can distinguish between the cuticular properties of these phenotypically distinct morphs of *G. mellonella*, melanic (tolerant) and non-melanic (susceptible). Upon encountering the NM larvae, *M. brunneum* activated an insect-specific attack strategy and proceeded with colonizing, penetrating and killing the host. Conversely, *M. brunneum* initiated a broader approach, e.g., plant-specific adhesion gene *Mad2*, when exposed to the tolerant M larvae, and demonstrated a reduced capacity to overcome the hosts’ preformed defenses. We contest that the cuticle surface of the M larvae is a nutrient poor, “stressful” environment leading to fewer fungi establishing themselves.

Collectively, these data enhance our understanding of insect-pathogen interactions, particularly with regards the intimate molecular traffic that make-up these “skirmishes on the cuticle” and versatile strategies used to outcompete each other. In response to distinct morphs of *G. mellonella, M. brunneum* demonstrates plasticity in gene expression and putative host range, which could be exploited for biocontrol efforts.

## Materials and methods

### Insects

Two separate geographic populations of the greater wax moth, *Galleria mellonella*, were used: the melanic (M) morph, and a non-melanic (NM) (Figure 1(a)). All insects were maintained at 28°C on an artificial medium (AM) as described in Dubovskiy et al., (2013) [12]. Wax moth larvae were photographed (in triplicate) under magnification (400x) with a Zeis Axio Imager A1 (Carl Zeiss, Germany). ImageJ 1.45 (National Institute of Health, USA) was used to quantify the extent of melanization and the size of melanic deposits on each cuticle (n = 10 per morph). The average cuticle thickness was calculated from the eighth sternite region of uninfected M (n = 35) and NM (n = 35) final instar larvae. Further details are provided in the Supplementary Materials S1.2.

### Fungal infections

*Metarhizium brunneum* ARSEF 4556 was used for all experiments. Unless otherwise stated, insects infected by topical application were final instar larvae raised in the same cohort, and sampled at 6, 12, 24 and 48-h post-infection (pi). Three topical doses of *M. brunneum* (1 × 10^4^, 1 × 10^5^ and 1 × 10^7^ conidia/ml) were used to determine the susceptibility of M and NM larvae to fungal infection over 12 d. To determine the resistance ratio (RR) of M and NM larvae, the LC_50_ of the M line was divided by the LC_50_ of the NM line. StatPlus 2009 (AnalystSoft Inc.) was used to conduct Probit analysis of the dose-mortality data to determine the LC_50_ value (i.e. concentration to kill 50% larvae), 95% fiducial limits of the LC_50_, the slope of the dose-mortality line, and the standard error of the slope. LC_50_ values were considered significantly different if their 95% fiducial limits did not overlap, which is a conservative criterion.

Topical exposure of M and NM *G. mellonella* larvae with two doses of *M. brunneum* conidia (1 × 10^5^ and 1 × 10^7^) were used to investigate insect and fungal gene expression (see the section below). The experimental sampling times reflected the optimal intervals to observe the acute stages of mycosis (when germination and penetration should be peaking in susceptible insects) and concomitant insect defences. Further details of fungus culture and inoculation methods are provided in the Supplementary Materials S1.3

### Conidial adhesion and germination

Conidial adhesion and germination on the surface of the M and NM larval cuticles were assessed using methods adapted from Ment et al. (2010) [51]. Further details are provided in the Supplementary Materials S1.4.

### Fatty acid *and hydrocarbon compositions of* G. mellonella *epicuticular waxes*

The fatty acid composition of the wax layer was determined using whole larvae before infection. Briefly, 3 independent groups of 20 larvae were immersed in 20 ml 99% dichloromethane and incubated in a rotary shaker (130 rpm) at 22°C for 5 min. Samples were dried under a stream of nitrogen before examination by GC-MS. Fatty acid methyl esters (FAMEs) were analyzed by an Agilent 6890GC interfaced directly to an Agilent 5975 mass spectrometer (split/splitless injection, 80:1 split ratio; 70eV, EI). The separation was achieved by a HP-INNOWAX (Agilent, USA) capillary column (30 m x 0.32 mm; film thickness 0.25 μm). The oven temperature program was held at 120°C for 9 min, and ramped at 20°C min^−1^ to 230°C where it was held for 10 min. The carrier gas was helium. Identification was achieved using the National Institute of Standards and Technology (NIST) spectral library and compared with published spectra. Quantification was achieved using a calibration curve of known amounts of C_17_ FAME using the same conditions as the samples. Peak areas were used for quantifying fatty acid and hydrocarbon compositions.

### Quantitative RT-PCR analysis of insect immunity-related and fungal virulence gene expression

To identify resistance factors, a comparison of the expression of genes was made in the integuments of M and NM larvae under native conditions (uninfected) and during fungal infection. Thirteen genes previously attributed to the immune response, repair, regeneration and stress regulation in *G. mellonella* were investigated [12,13]: antimicrobial peptides (gallerimycin, gloverin), the multi-functional protein apolipophorin III, siderophores (transferrin and ferritin), the insect metalloproteinase inhibitor (IMPI), heat-shock protein 90 (HSP-90), oxidative stress (peroxidase (contig 17,373) and glutathione-S-transferase (GST)); cell proliferation (Contigs 704 and 233), and two enzymes involved in melanin synthesis (prophenoloxidase (PPO) and DOPA-decarboxylase (DCC)) .

Comparisons of fungal virulence and stress management gene expression were made across both M and NM larvae after topical exposure (i.e., the presence of fungi on the integument). Eight genes previously detected as a part of virulence and stress regulation in *Metarhizium sp*. were investigated [12,13]. Such genes code for Subtilisin-like proteases (Pr1a/b and Pr2), heat-shock proteins (HSP30 and HSP70), adhesin-like proteins (MAD1, MAD2), one multifactorial transcription factor cag 8 (responsible for hydrophobin synthesis and mycelial growth), and one nitrogen regulator (nrr) [3].

Gene expression (mRNA) was measured by quantitative, reverse transcriptase PCR using normalized cDNA samples with an CFX96 Touch™ Real-Time PCR Detection System (Bio Rad, USA) relative to reference genes, *Elongation Factor 1-alpha* (EF1; AF423811) for insects and *Translation elongation factor 1-alpha* (tEF; XM_014686196.1) for fungi [52]. Reactions were prepared by following the manufacturer’s protocol for the Rotor-Gene SYBR Green PCR mix (Qiagen). Further details are provided in Supplementary Materials S1.6 and S1.7, SI Table 1 and SI Table 2.

## Data analyses

Data were analyzed using GraphPad Prism v7.0 (GraphPad Software Inc, USA). Data were checked for normality (Gaussian) using the D’Agostino-Pearson omnibus test, and if non-normally distributed, a more conservative non-parametric analysis was applied. For qRT-PCR data with a Gaussian distribution, Grubbs’ extreme studentized deviate (ESD) test was used to exclude extreme outliers. Triplicate samples comprising integuments from five insects were used for genes expression (qRT-PCR analysis). Adhesion of conidia, cuticular thickness, melanization and size of melanic spots, epicuticular hydrocarbons and fatty acids comparisons between M and NM morphs were made using an unpaired t-test. Germination and the number of conidia detected on cuticles were compared using two-way ANOVA (with Bonferroni post hoc tests). Individual gene comparisons were made with non-parametric one-way ANOVA (Kruskal–Wallis with Dunn’s post hoc test). Cox’s proportional hazards and survival regression was used to assess differences in mortality rates after fungal infections between M and NM larvae.

## Supplementary Material

Supplemental Material

## Datasets are available on request

The raw data supporting the conclusions of this manuscript will be made available by the authors (ID and EG), without undue reservation, to any qualified researcher.
