# Supplementary material for: Metarhizium brunneum infection dynamics differ at the cuticle interface of susceptible and tolerant morphs of Galleria mellonella
Source: Virulence. 2019 Nov 25;10(1):999–1012. doi: 10.1080/21505594.2019.1693230 (PMC8647853; doi:10.1080/21505594.2019.1693230)
Supplement: Supplemental Material [file KVIR_A_1693230_SM8323.docx]

Supplementary information

***Metarhizium brunneum* infection dynamics differ at the cuticle interface of susceptible and tolerant morphs of *Galleria mellonella***

Ekaterina V. Grizanova^1^, Christopher J. Coates^2^*, Ivan M. Dubovskiy^1,3^, Tariq M. Butt^2,^

##### MATERIALS AND METHODS

#### S1.1: Reagents and buffers

Bovine serum albumin (BSA), phenylthiourea (PTU), ethylenediaminetetraacetic acid (EDTA) and 3,4-dihydroxy-L-phenylalanine (DOPA) were all supplied by Sigma–Aldrich, UK. Anticoagulant (AC) comprised 62 mM NaCl, 100 mM glucose, 10 mM EDTA, 30 mM sodium citrate and 26 mM citric acid (pH 4.6). All solutions were made in pyrogen-free ultra-pure water.

#### S1.2: Insect rearing

The melanic (M) dark morph and non-melanic (NM) morph of *G. mellonella* were reared in strict isolation at 28°C, 60% relative humidity, with a 12:12h light:dark cycle, and fed on artificial medium (AM) containing 22.5% corn meal, 12.5% honey, 12.5% glycerol, 12.5% beeswax, 10% wheat flour, 12.5% milk solids, 5% yeast and 12.5% water. The M morph originated from Western Siberia, and was collected from private apiaries within a 5-10km radius of each other in the Novosibirsk region (latitude 54°21 longitude 77°17) in 2005, where it was found in normal population densities (i.e. it was not overcrowded). The M insects were maintained under laboratory conditions at the Laboratory of Biological Plant Protection and Biotechnology, Novosibirsk State Agrarian University. It is currently unknown whether the dark insect morph is genetically dominant. **The** NM morph originated from Europe and was maintained under laboratory conditions for 10 years at Swansea University, UK.

**S1.3: Fungus cultivation and infections**

*Metarhizium brunneum* was grown on Sabouraud’s dextrose agar (SDA) at 25°C for 14 d. Conidia were harvested by scraping from sporulating cultures, air-dried at RT for 1 week and stored at 4°C. For topical infections, conidia were suspended in sterile 0.03% Tween–80 and vortexed for 1 min. Viability of conidia was verified by incubation of these propagules on SDA and determining the percentage germination. Only suspensions with at least 99% germination were used.

Natural (topical) application of conidia was achieved by a single 10 s dip of each insect in a 0.03% Tween 80 (v/v) suspension of conidia. Uninfected control larvae were dipped in 0.03% Tween 80 only. Dipped insects were kept in Petri dishes (10 larvae / dish) with 3g artificial medium. For survival assays, larvae were observed daily for <12 days (depending on the experiment) and all dead animals were removed and examined to determine the cause of death.

**S1.4: Conidial adhesion and germination**

Conidial adherence and germination was studied using methods adapted from [[1](#_ENREF_1),[2](#_ENREF_2)]. Following 6 h topical inoculation, non-adhered conidia were washed from the larvae with 0.01% Triton X-100 and the larvae rinsed with water. Each larva was then placed in a glass tube with 4 ml of 99% dichloromethane and shaken vigorously (250 rpm) for 10 min to detach the adhered conidia from the cuticle. The dichloromethane was decanted into 4 ml 99% ethanol and the conidia pelleted by centrifugation at 3000g for 30 min at 25°C. After evaporation of the solvent, the conidia were suspended in 0.05% Tween-80 and the concentration determined using a haemocytometer.

Determination of the extent of *in vivo* conidial germination on live larval cuticle was performed by topical application of the fungus as above, and at 6 h, 12 h, 24 h and 72 h, whole larvae were fixed in 2% formaldehyde. Before microscopial investigation, larvae were dipped in an aqueous solution of 0.1% Calcofluor white stain for 25 s [[3](#_ENREF_3)]. The larvae were then air-dried and the head and haemocoel contents were removed. The cuticle was observed using an Zeiss Axio Imager.A1 fluorescent microscope (Carl Zeiss, Germany). The stain allows the visualization of fungal cell walls so that it is possible to identify and count fungal germ tubes and hyphal penetration events. To establish the germination level, about 100 germinated conidia were observed for each larva. Larvae on which more than 50% of conidia had germinated were considered to “germination positive”. Larvae were considered to be “penetration positive” when penetrating hyphae were observed in ten or more sites on the cuticle (with active hyphal penetration / invasion and evidence of localized melanin formation).

###### S1.5: Cuticle thickness

Final instar larvae, 3 days after moulting, were frozen at -20°C and the 8th sternite region was sectioned with a Microm HM 520 microtome. Slices were then chemically fixed onto glass slides. Three thickness measurements were made in each of five sections per larva, and the calculated average thickness for each of 20 M and NM larvae were compared. Measurements were made with an Axioscope 40 microscope and analysed using AxioVision 40 (v4.6.3.0) software.

####

#### S1.6: Preparation of insect integuments, RNA extractions and cDNA synthesis

Uninfected larvae and larvae at 6h, 12h, 24h, 48h and 72h after topical infection with *Metarhizium brunneum* were sampled. At each time point non-adhered conidia were washed from the larvae by vortexing for 10 sec in 0.01% Triton X-100 and the larvae rinsed with water. Integuments were dissected in PBS in sterile condition to avoid contamination, than stored in RNA-later (Ambion), and then freeze-dried before RNA extraction. Freeze-dried samples of integuments were homogenized in 1 ml of TRIzol® Reagent (Invitrogen) with glass beats (soil grinding SK38, Precellys® Lysing Kit, Bertin) with a FastPrep®-24 homogenizer (MP Biomedicals, USA) 2 cycles for 30 sec at 6.5 M/s, with cool down in ice for 30 sec between cycles of homogenization. The homogenates were centrifuged for 10 min at 15000xg at 4 °C. Total RNA was isolated using Pure Link RNA Mini Kit (Ambion) according to the manufacturer’s recommendations. Digestion of DNA was performed on-column with PureLink™ DNase Set (Invitrogen) during critical RNA purification procedures. RNA concentrations were determined spectrophometrically and cDNA synthesis was performed with 1μg RNA using qScript™ cDNA synthesis kit (Quanta Bioscience) by following the manufacturer’s protocol.

**S1.7: QRT-PCR assay for expression of insect genes**

cDNA quantity was checked and normalised using reference gene PCR of 1/50 dilutions of each sample measured against a standard curve, and sufficient cDNA of similar concentration for each sample diluted to amplify all genes. Samples were quality checked for consistency between values for the reference gene used, *Elongation Factor 1-alpha* (*EF1*) (AF423811). Expression was then measured in the normalised samples using the CFX96 Real-Time PCR detection system (Biorad, USA). Primers were designed from published *G. mellonella*sequences (NCBI) or from coding sequence where high homology protein sequences could be identified from an EST library [[4](#_ENREF_4)] and are given in Table S1. For *HSP90*, a primer designed to the conserved 3’UTR region found in Lepidoptera [[5](#_ENREF_5)] was paired with a degenerate primer designed from an alignment of 8 lepidopteran *HSP90* sequences (GU230738, AB214972, AB060275, EF197936, GU230737, AF254880, GU230739, AB206477) using CODEHOP [[6](#_ENREF_6)]. Other primers were designed using PerlPrimer [[7](#_ENREF_7)] or Primer3 [[8](#_ENREF_8)] to amplify at 60˚C with an amplicon size of 80-200bp, rechecked for potential dimer formation with Oligo 6 (Molecular Biology Insights, Inc, http://oligo.net/), and for amplicon secondary structure using the Mfold server [[9](#_ENREF_9)]. Primers were optimised by checking products for a clean single peak by high resolution melt curve (HRM) analysis and by titrating concentration for optimal efficiency using a serial dilution of mixed cDNA, and were redesigned if necessary to produce a clean PCR product.

A mix of 5 μl Rotor-Gene SYBR Green PCR mix (Qiagen) and 1 μl of primers mix was added to 4 μl cDNA for each 10 μl PCR. Cycling conditions were 95˚C 5 min followed by 42 cycles of: 95˚C 10 sec, annealing 15sec, 72˚C 30 sec. An initial touchdown of 1˚C per cycle from 65˚C for the first 5 cycles. HRM analysis performed at the end of each run allowed each PCR to be checked for the presence of the expected product. All reactions were performed in triplicate, and optimal threshold values and reaction efficiencies calculated from 7-point serial dilutions of mixed cDNA from fungal infected insects. Fold change values were calculated using the ΔΔCt method: for each locus, the ΔΔCt for sample was determined by subtracting the measured Ct value from the Ct value of reference (‘housekeeping’) *EF1* gene (listed in Table SX). ΔΔCts were then converted to relative copy numbers using the formula, 2^Δ^ΔΔ^Ct^. [[10](#_ENREF_10)].

**S1.8: QRT-PCR assay for expression of fungi genes**

cDNA quantity was checked and normalised using reference gene PCR of 1/5 dilutions of each sample measured against a standard curve, and sufficient cDNA of similar concentration for each sample diluted to amplify all genes. Samples were quality checked for consistency between values for the reference genes, *Translation elongation factor 1-alpha* (tEF). Expression was then measured in the normalised samples using the CFX96 Real-Time PCR detection system (Biorad). Primers were optimised by checking products for a clean single peak by high resolution melt curve (HRM) analysis and by titrating concentration for optimal efficiency using a serial dilution of mixed cDNA, and were redesigned if necessary to produce a clean PCR product.

A mix of 5 μl Rotor-Gene SYBR Green PCR mix (Qiagen) and 1 μl of primers mix was added to 4 μl cDNA for each 10 μl PCR. For *M. brunneum* genes, *HSP*30 and *Pr1*B cycling conditions 45˚C 5 min, 95˚C 3 min, and 39 cycles of 95˚C 10 sec, 54˚C 10 sec and 72˚C 30 sec was used. For M. brunneum gene HSP70 cycling conditions 45˚C 5 min, 95˚C 3 min, and 39 cycles of 95˚C 10 sec, 55˚C 10 sec and 72˚C 30 sec was used. For M. brunneum genes Pr2, Pr1A, Mad2 and nrr1 cycling conditions 45˚C 5 min, 95˚C 3 min, and 39 cycles of 95˚C 10 sec, 56˚C 10 sec and 72˚C 30 sec was used. For M. brunneum gene tEF and Cag8 cycling conditions 45˚C 5 min, 95˚C 3 min, and 39 cycles of 95˚C 10 sec, 57˚C 10 sec and 72˚C 30 sec was used. HRM analysis performed at the end of each run allowed each PCR to be checked for the presence of the expected product. All reactions were performed in triplicate, and optimal threshold values and reaction efficiencies calculated from 7-point serial dilutions of mixed cDNA from fungal infected insects. Fold change values were calculated using the ΔΔCt method: for each locus, the ΔΔCt for sample was determined by subtracting the measured Ct value from the Ct value of the reference gene t*EF* (listed in Table SX). ΔΔCts were then converted to relative copy numbers with the formula 2^Δ^ΔΔ^Ct^ [10].

**S2. SUPPLEMENTARY REFERENCES**

1. Ment D, Gindin G, Rot A, Soroker V, Glazer I, et al. (2010) Novel Technique for Quantifying Adhesion of Metarhizium brunneum Conidia to the Tick Cuticle. Applied and Environmental Microbiology 76: 3521-3528.

2. Ment D, Gindin G, Soroker V, Glazer I, Rot A, et al. (2010) Metarhizium brunneum conidial responses to lipids from tick cuticle and tick mammalian host surface. Journal of Invertebrate Pathology 103: 132-139.

3. Butt TM (1997) Complementary Techniques: Fluorescence Microscopy. In: Lacey LA, editor. Manual of Techniques in Insect Pathology. London: Academic Press. pp. 255-365.

4. Vogel H, Altincicek B, Glockner G, Vilcinskas A (2011) A comprehensive transcriptome and immune-gene repertoire of the lepidopteran model host Galleria mellonella. BMC Genomics 12: 308.

5. Xu PJ, Li T, Xiao JH, Murphy RW, Huang DW (2011) Universal primers for amplifying the complete coding sequence of cytoplasmic heat shock protein 90 (HSP90) in Lepidoptera. European Journal of Entomology 108: 164-168.

6. Staheli JP, Boyce R, Kovarik D, Rose TM (2011) CODEHOP PCR and CODEHOP PCR primer design. Methods Mol Biol 687: 57-73.

7. Marshall OJ (2004) PerlPrimer: cross-platform, graphical primer design for standard, bisulphite and real-time PCR. Bioinformatics 20: 2471-2472.

8. Rozen S, Skaletsky H (2000) Primer3 on the WWW for general users and for biologist programmers. Methods Mol Biol 132: 365-386.

9. Zuker M (2003) Mfold web server for nucleic acid folding and hybridization prediction. Nucleic Acids Research 31: 3406-3415.

10. Pfaffl MW (2001) A new mathematical model for relative quantification in real-time RT-PCR. Nucleic Acids Res 29: e45.

**Supplementary Table 1 Insect genes used for (mRNA) expression analysis**

| **Genes** | **Reference** | **Description** | **Oligonucleotides** |
| --- | --- | --- | --- |
| EF1: Elongation factor 1-alpha | AF423811 | Housekeeping (reference gene) | For: AACCTCCTTACAGTGAATCC  Rev: ATGTTATCTCCGTGCCAG |
| Gallerimycin  [antifungal peptide] | AF453824 | Antifungal peptide  Schuhmann et al. (2003) | For: GAAGTCTACAGAATCACACGA  Rev: ATCGAAGACATTGACATCCA |
| Gloverin-like protein  [antibacterial peptide] | AF394588 | Antibacterial peptide  Hwang and Kim (2011) | For: AGATGCACGGTCCTACAG  Rev: GATCGTAGGTGCCTTGTG |
| Transferrin precursor (TSF) | AY364430 | Siderophore/Antimicrobial | For: CGTAGCAGTCATCAAGAAGG  Rev: CGCACTCACTAGAACTGG |
| Insect metalloproteinase inhibitor  (IMPI) | AY330624 | Inhibitor of microbial metalloproteinase; Wedde et al. (2007) | For: TAGTAAGCAGTAGCATAGTCC  Rev: GCCATCTTCACAGTAGCA |
| Contig 17373  [phospholipid hydroperoxide] | contig17373_1.exp  Vogel et al. (2011) | Glutathione peroxidase activity | For: CCACACTGTGAGGCAACATT  Rev: GTTTGCTTAGCACGGTCACA |
| Contig 704  [pleiotrophin-like protein] | GME-string_Contig_704.0  Vogel et al. (2011) | Putative mitogen | For: GAGGTGCCTGGTCAGAATGT  Rev: TGGGGTCACTATTCGCTTTC |
| Contig 233  [growth-blocking peptide] | GME-string_Contig_233.0  Vogel et al. (2011) | Immunity/cell proliferation  Tsuzki et al (2012) | For: CGTGACGAGTGAGATCGTTG  Rev: CGGTTGTTGTCTCCGTTACA |
| Heat shock protein 90 (HSP 90) | Xu et al. (2011) | Stress management [chaperone] | For: GCRTCVCGYATGGAGGAAGT  Rev: GAACTAAATCAGTCTTTGG |
| Apolipophorin III  [anti-fungal factor] | This study | Immunity/Fungal recognition  Whitten et al (2001); Taszlow and Wojda (2015) | For: CGTTCACCAACTCGAAGGACA  Rev: GCGCTTTCTGCAAACTGGA |
| Ferritin  [siderophore] | This study | Oxidative stress/immunity  Pham and Winzerling (2010) | For: TGCTTCCTCGCCGTGTCTG  Rev: TGCATCTCTGTGGCGACGTT |
| Glutathione-S-transferase (GST) | This study | Detoxification  (REDOX balance) | For: CTTCGATATTGGCACCCTGT  Rev: TTCTCAGCCCTCGTTCACTT |
| proPhenoloxidase (PPO) | This study | Immunity – melanin synthesis  Whitten and Coates (2017) | For: TGTCCAATCC GCCGCAGTTT CCTA  Rev: CGCCAATAAG CAAGACGGTG TTCC |
| DOPA decarboxylase (DDC) | This study | Immunity – melanin synthesis  Whitten and Coates (2017) | For: GCTAACTCGTATCCGTCTATCGT  Rev: CCTCCAGCTCAGTACATGCC |

**References**

- Vogel H, Altincicek B, Glockner G, Vilcinskas A (2011) A comprehensive transcriptome and immune-gene repertoire of the lepidopteran model host Galleria mellonella. BMC Genomics 12: 308.
- Xu PJ, Li T, Xiao JH, Murphy RW, Huang DW (2011) Universal primers for amplifying the complete coding sequence of cytoplasmic heat shock protein 90 (HSP90) in Lepidoptera. European Journal of Entomology 108: 164-168.
- Schuhmann B, Seitz V, Vilcinskas A, Podsiadlowski L. (2003) Cloning and expression of gallerimycin, an antifungal peptide expressed in immune response of greater wax moth larvae, Galleria mellonella. Arch Insect Biochem Physiol. Jul;53(3):125-33.
- Wang Q, Liu Y, He HJ, Zhao XF, Wang JX. (2010) Immune responses of Helicoverpa armigera to different kinds of pathogens. BMC Immunol. 3;11:9.
- Hwang J, Kim Y. (2011) RNA interference of an antimicrobial peptide, gloverin, of the beet armyworm, Spodoptera exigua, enhances susceptibility to Bacillus thuringiensis. J Invertebr Pathol. 108(3):194-200.
- Ekengren S, Hultmark D. (1999) Drosophila cecropin as an antifungal agent. Insect Biochem Mol Biol. 29(11):965-72.
- Cytryńska M, Mak P, Zdybicka-Barabas A, Suder P, Jakubowicz T. (2007) Purification and characterization of eight peptides from Galleria mellonella immune hemolymph. Peptides. 28(3):533-46.
- J.H. Lee , S. Park , K.-S. Chae, I.H. Lee (2010) Galleria mellonella 6-Tox Gene, Putative Immune Related Molecule in Lepidoptera Int. J. Indust. Entomol. Vol. 21, No. 1, pp. 127-132
- T. Yoshiga , T. Georgieva , B. C. Dunkov , N. Harizanova , K. Ralchev, J. H. Law (1999) Drosophila melanogaster transferrin Cloning, deduced protein sequence, expression during the life cycle, gene localization and up-regulation on bacterial infection Eur. J. Biochem. 260, 414-420
- Wedde M, Weise C, Nuck R, Altincicek B, Vilcinskas A. (2007) The insect metalloproteinase inhibitor gene of the lepidopteran Galleria mellonella encodes two distinct inhibitors. Biol Chem. 388(1):119-27.
- Dubovskiy I.M., Whitten M.M.A., Yaroslavtseva O.N., Greig C., Kryukov V.Y., Grizanova E.V., Mukherjee K., Vilcinskas A., Glupov V.V., Butt T.M. (2013) Can insects develop resistance to insect pathogenic fungi? PLoS ONE 8(4): e60248.
- S. Tsuzuki, M. Ochiai, H. Matsumoto, S. Kurata, A. Ohnishi, Y. Hayakawaa, (2012) Drosophila growth-blocking peptide-like factor mediates acute immune reactions during infectious and non-infectious stress Sci Rep.; 2: 210.
- Whitten, M. M., Tew, I. F., Lee, B. L., & Ratcliffe, N. A. (2004). A novel role for an insect apolipoprotein (apolipophorin III) in β-1, 3-glucan pattern recognition and cellular encapsulation reactions. *The Journal of Immunology*, *172*(4), 2177-2185.
- Taszlow, P., Wojda, I., 2015. Сhanges in the hemolymph protein profiles in Galleria mellonella infected with Bacillus thuringiensis involve apolipophorin III. The effect of heat shock. Archives of insect biochemistry and physiology, 88, 123-143.
- Pham, D. and Winzerling J. 2010. Insect Ferritins: typical or atypical? Biochim Biophys Acta. 2010, 1800(8): 824–833. doi:10.1016/j.bbagen.2010.03.004.
- Whitten, M. M., & Coates, C. J. (2017). Re‐evaluation of insect melanogenesis research: Views from the dark side. *Pigment cell & melanoma research*, *30*(4), 386-401.

**Supplementary Table 2 Fungal genes used for (mRNA) expression analysis**

| **Genes** | **Description** | **Oligonucleotides** |
| --- | --- | --- |
| tEF; Translation elongation factor | Housekeeping (reference gene)  Butt et al. (2013); Fang and Bidochka (2006) | For: CGAGCGTGAGCGTGGTA  Rev: CAGCCTCGAACTCACCAG |
| Pr1**a** | Subtilisin-like protease – cuticle degradation  Stegler et al. (1994 and 1995) | For: TCCGAGTCCTCTTGCCTATCT  Rev: GGCACCGTTGTAGGCAAGGTAGTT |
| Pr1**b** | Subtilisin-like protease – cuticle degradation  Stegler et al. (1994 and 1995) | For: ATCTAAGACGCCATCCTGAC  Rev: ATGTATGAGCATGTTCCTTCG |
| Pr2 | Trypsin-like protease – cuticle degradation  Stegler et al. (1994 and 1995) | For: TACGCCACATTGCCAGAG  Rev: GCATGTCGCACGATCAAC |
| Heat shock protein 30 (HSP30) | Stress management [chaperone]  Liao et al. (2014) | For: GGTCCAACGCATCACACT  Rev: CTTCTTCTCCTCGGGCTCA |
| Heat shock protein 70 (HSP70) | Stress management [chaperone]  Liao et al. (2014) | For: CTGTCAACAATGCCGTCATCA  Rev: ATATCATCCTTGTCCTTGTCCTC |
| Mad1 | Adhesion to cuticle (virulence)  Barelli et al. (2011); Wang and St Leger (2007) | For: CTCCTCACATCACCCAGGTT  Rev: GGGAGTAGGCATGACGATGT |
| Mad2 | Adhesion to plant (virulence)  Barelli et al. (2011); Wang and St Leger (2007) | For: CTATGTCCACCCTTGCGACT  Rev: AGCACAGCTGATGAGGGTCT |
| Conidiation-associated gene (Cag 8)  [regulator of –protein signalling] | Multifactorial: hydrophobin synthesis, mycelial growth, blastospores production, regulation of conidiation  Fang et al. (2007) | For: AAGCTGATGGCTAGCGATTC  Rev: TTGCGGTTGGAACGACTTTG |
| Nitrogen response regulator (nrr) | Nutrient assimilation  Screen et al. (1998) | For: AGTATTGATGAGCGTCGTAAC  Rev: TGCGTCGTTGTCCATGAAG |

**References**

- Stleger, R. J., Bidochka, M. J., and Roberts, D. W. (1994). Isoforms of the Cuticle-Degrading Pr1 Proteinase and Production of a Metalloproteinase by Metarhizium-Brunneum. Archives of Biochemistry and Biophysics 313, 1-7.
- Stleger, R. J., Joshi, L., Bidochka, M. J., and Roberts, D. W. (1995). Protein-Synthesis in Metarhizium-Brunneum Growing on Host Cuticle. Mycological Research 99, 1034-1040.
- Liao, X., Lu, H. L., Fang, W., and St Leger, R. J. (2014). Overexpression of a *Metarhizium robertsii* HSP25 gene increases thermotolerance and survival in soil. Appl Microbiol Biotechnol 98, 777-83.
- Barelli, L., Padilla-Guerrero, I. E., and Bidochka, M. J. (2011a). Differential expression of insect and plant specific adhesin genes, Mad1 and Mad2, in *Metarhizium robertsii*. Fungal Biol 115, 1174-85.
- Fang, W., & Bidochka, M. J. (2006). Expression of genes involved in germination, conidiogenesis and pathogenesis in Metarhizium brunneum using quantitative real-time RT-PCR. *Mycological research*, *110*(10), 1165-1171.
- Fang, W., Pei, Y., and Bidochka, M. J. (2007). A regulator of a G protein signalling (RGS) gene, cag8, from the insect-pathogenic fungus Metarhizium brunneum is involved in conidiation, virulence and hydrophobin synthesis. Microbiology 153, 1017-25.
- Screen, S., Bailey, A., Charnley, K., Cooper, R., and Clarkson, J. (1998). Isolation of a nitrogen response regulator gene (nrr1) from Metarhizium brunneum. Gene 221, 17-24.
- Butt TM, Greenfield BPJ, Greig C, Maffeis TGG, Taylor JWD, et al. (2013) Metarhizium brunneum Pathogenesis of Mosquito Larvae: A Verdict of Accidental Death. PLoS ONE 8(12): e81686. doi:10.1371/journal.pone.0081686
- Wang, C., & St Leger, R. J. (2007). The MAD1 adhesin of *Metarhizium brunneum* links adhesion with blastospore production and virulence to insects, and the MAD2 adhesin enables attachment to plants. *Eukaryotic cell*, *6*(5), 808-816.


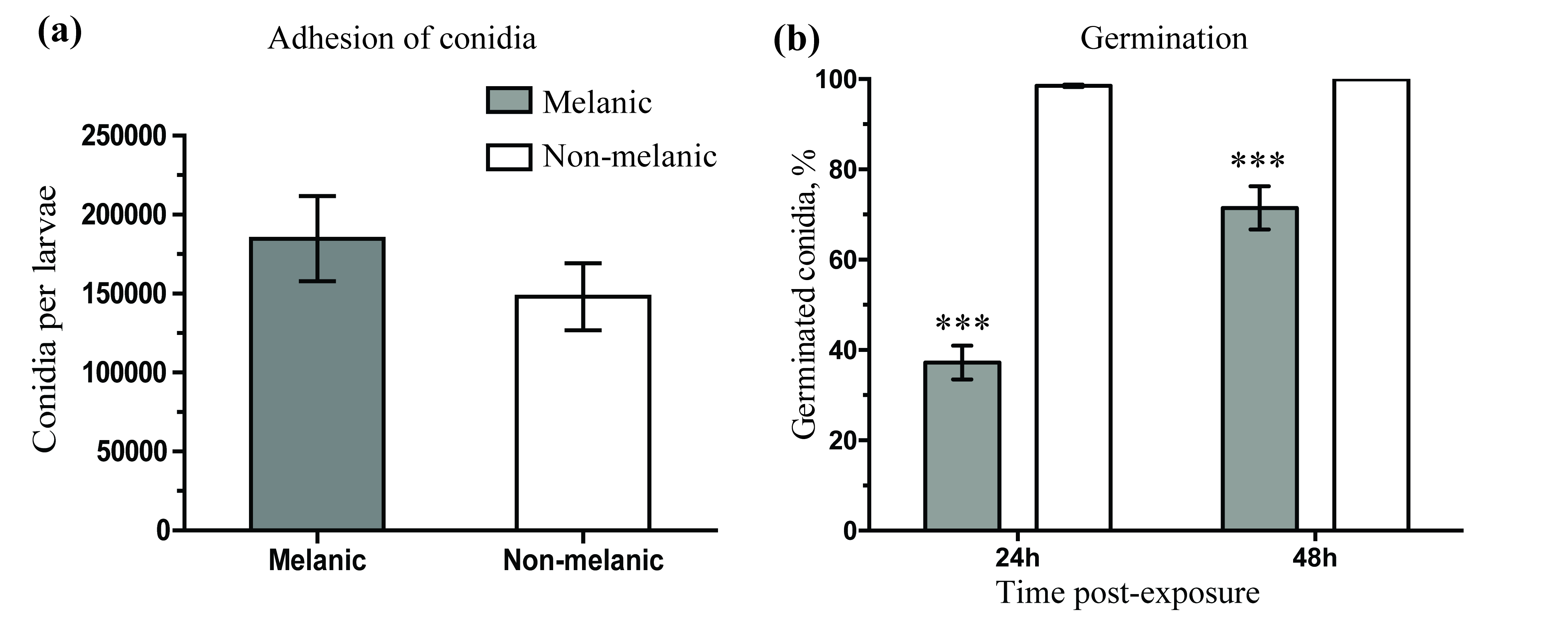


**Supplementary Figure 1** Adhesion (a) and germination (b) of conidia on M and NM larvae cuticle (***p<0.001 compare with NM for same time point).
